# Accuracy Evaluation of Circular RNA in Diagnosing Lung Cancer in a Chinese Population

**DOI:** 10.1155/2019/7485389

**Published:** 2019-10-20

**Authors:** Zhihao Xiao, Xinglei Chen, Xiaodan Lu, Xuexin Zhong, Yihui Ling

**Affiliations:** Institute for Chemical Carcinogenesis, Guangzhou Medical University, Guangzhou, China

## Abstract

Circular RNA (circRNA) is a class of recently discovered noncoding RNA. circRNAs can be used as a potent noninvasive biological marker of cancer owing to their varying expression levels among different cancers. This meta-analysis was performed to assess the accuracy of circRNAs in diagnosing lung cancer. A total of eight studies identified through searching the PubMed, Web of Science, Cochrane Library, and Embase from inception to March 20, 2019 were eligible for this meta-analysis. The pooled sensitivity, specificity, positive likelihood ratios, negative likelihood ratios, and diagnostic odds ratio were 0.77 (95% confidence interval (CI): 0.73–0.80; *I*^2^ = 8.98%), 0.76 (95% CI: 0.69–0.82; *I*^2^ = 63.12%), 3.17 (95% CI: 2.43–4.14; *I*^2^ = 33.18%), 0.31 (95% CI: 0.26–0.37; *I*^2^ = 20.36%), and 10.26 (95% CI: 6.87–15.31; *I*^2^ = 97.18%), respectively. The area under the receiver operating characteristic curve was 0.78 (95% CI: 0.74–0.81). The study confirmed the use of circRNAs in diagnosing lung cancer in a Chinese population.

## 1. Introduction

Circular RNA (circRNA) is a circular single-strand RNA first discovered in plants [1, 2] and abundant in human cells. In some cases, the abundance of circRNAs exceeds that of associated linear mRNAs by more than tenfold [3]. CircRNAs lack free ends, 5′ cap, and 3′ poly(A) tail and are more stable than linear RNAs with their ends joining in a circle via phosphodiester bonds [4]. CircRNAs have many functions including regulating gene transcription and translation via binding to miRNAs, interacting with proteins, and being directly translated [5]. Recent studies suggested significant advantages of circRNAs in diagnosing cancers owing to their prevalence, stability, specificity, and conservatism [6].

Experiments proved that circRNAs were associated with proliferation, apoptosis, invasion, and migration of tumor cells, and the expression varied with tumor cells, thus helping in diagnosing and predicting the prognosis of tumors. Li et al. [7] found that Hsa_circ_0000096 was significantly downregulated in gastric cancer tissues and cell lines and was associated with the invasion and staging of tumors. Ma et al. [8] reported that circRNA-000284 was significantly higher in cervical cancer cells than in cervical epithelial cells and could serve as a biological marker. Li et al. [9] considered circHIPK3 as the novel therapeutic target of bladder carcinoma. Some meta-analyses summarizing the role of circRNAs on diagnosis and prognosis were reported [10–13]. Huang et al. [10] investigated the prognostic and diagnostic significance of the expression of circRNAs in patients with hepatocellular carcinoma. Wang et al. [11], Li et al. [12], and Ding [13] performed a meta-analysis on the value of circRNAs as a biological marker of tumors. However, they just explored the role of circRNAs in diagnosing tumors but not specially or independently diagnosing lung cancer.

Lung cancer accounts for 25% of cancer-related deaths worldwide, which is much higher compared with other cancers such as breast carcinoma, prostatic carcinoma, and colorectal carcinoma. The survival rate of patients with lung cancer is relatively low, possibly due to the lack of early testing methods [14]. The role of circRNAs in lung cancer was first reported in 2018 by Qu et al. [5] who found that hsa_circ_00013958 promoted the proliferation, invasion, and metastasis of lung adenocarcinoma cells. A large number of recent studies explored the relationship between circRNAs and lung cancer [15–21].

## 2. Materials and Methods

### 2.1. Literature Search

Electronic databases, including PubMed, Web of Science, Cochrane Library, and Embase, were systematically searched from inception to March 20, 2019. The following search terms were used: circRNA, circular RNA, lung cancer, lung neoplasm, lung adenocarcinoma, non-small-cell lung cancer, NSCLC, pulmonary cancer, and pulmonary neoplasm. In addition, the reference lists of eligible studies were manually searched to guarantee the comprehensiveness of the literature.

### 2.2. Inclusion and Exclusion Criteria

The inclusion criteria were as follows: (1) studies analyzing the relationship between circRNAs and lung cancer, (2) studies providing data on the sensitivity and specificity, and (3) studies involving ≥30 patients and controls. The exclusion criteria were as follows: (1) repetitive research; (2) letters, editorials, commentaries, or abstracts; (3) studies involving ineligible patients or controls; (4) studies lacking data; or (5) studies in a non-English language. If the results came from the overlapping population, only the first study or the most complete study was included.

### 2.3. Data Extraction and Quality Assessment

Two reviewers (Xuexin Zhong and Zhihao Xiao) extracted data. The discrepancies were resolved by the third reviewer (Xiaodan Lu) if needed. The following information was extracted from each study: first author name, year of publication, country, sample size, sample type, sensitivity, specificity, area under the receiver operating characteristic (ROC) curve (AUC), testing methods, tumor staging, reference gene, and differential expression of circRNAs.

### 2.4. Statistical Analysis

Stata 12.0 software and Meta-DiSc 1.4 were used for statistical analysis. The sensitivity, specificity, positive likelihood ratio (PLR), negative likelihood ratio (NLR), diagnostic odds ratio (DOR), 95% confidential intervals (95% CI), summary ROC (SROC) curve, and AUC were calculated for quality assessment. The significance level was set as *P* < 0.05. The heterogeneity induced by the threshold effect of included studies was tested using the Spearman correlation analysis. Cochran's *Q* and *I*^2^ tests were used to assess the heterogeneity of data. *I*^2^ > 50% indicated significant heterogeneity. A subgroup analysis was performed based on sample type, cancer type, reference gene type, and differential expression of circRNAs. The potential source of heterogeneity by the nonthreshold effect was analyzed by regression analysis. A Fagan nomogram was used to calculate the posttest probabilities. Finally, publication bias was assessed.

## 3. Results

### 3.1. Search Results

**Figure 1:**
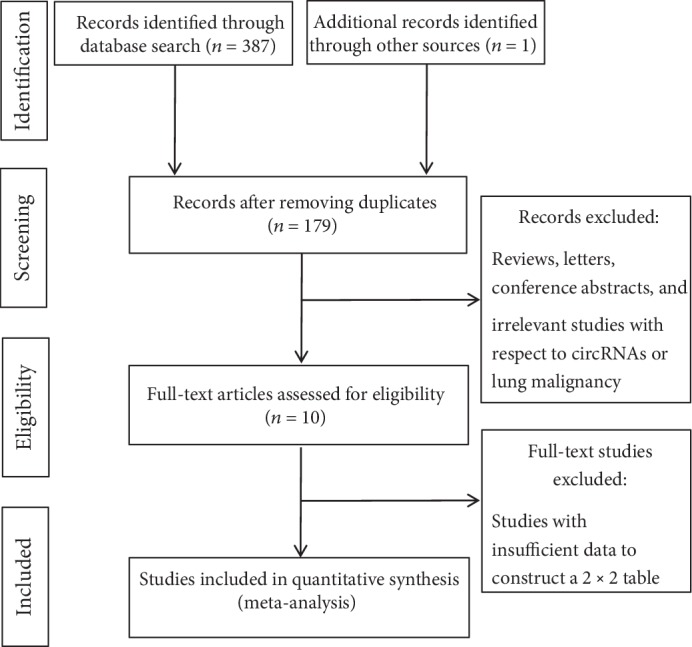
Flow chart of the study selection process.

The flowchart of the study selection process is shown in Figure 1. The review of the literature identified 388 studies, of which 209 repeated ones were excluded and the remaining 179 ones were screened based on titles and abstracts. Subsequently, 165 studies, including reviews, letters, conference abstracts, and 2 studies on hsa_circ_0102533 [22] and circFARSA [23], were further excluded, owing to the inability of constructing a 2 × 2 contingency table. A total of 8 studies [15–21, 24] and 10 eligible studies, involving 668 patients with lung cancer and 153 healthy controls, were assessed during this meta-analysis. The characteristics of eight studies are shown in Table 1. Surprisingly, all eight studies identified using search terms, inclusion criteria, and exclusion criteria were performed in China.

### 3.2. Threshold Effect

The threshold effect was evaluated with the Spearman rank correlation. The Spearman correlation coefficient was 0.079 (*P* = 0.829), suggesting no threshold effect.

### 3.3. Results of Meta-Analysis

**Figure 2:**
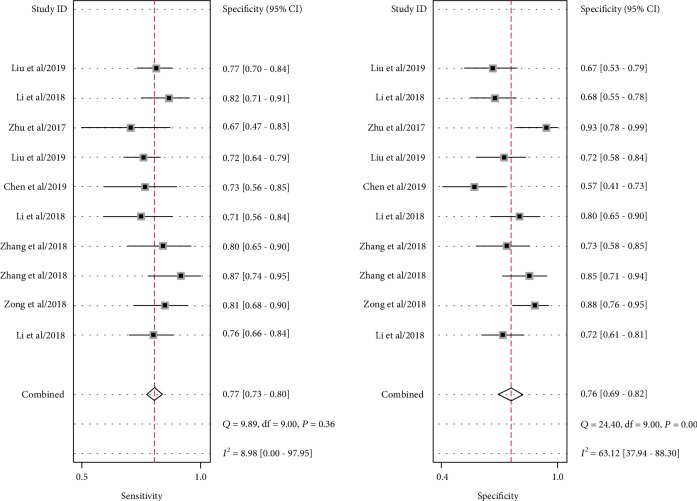
Forest plots of the sensitivity and specificity for circRNAs in diagnosing lung cancer.

**Figure 3:**
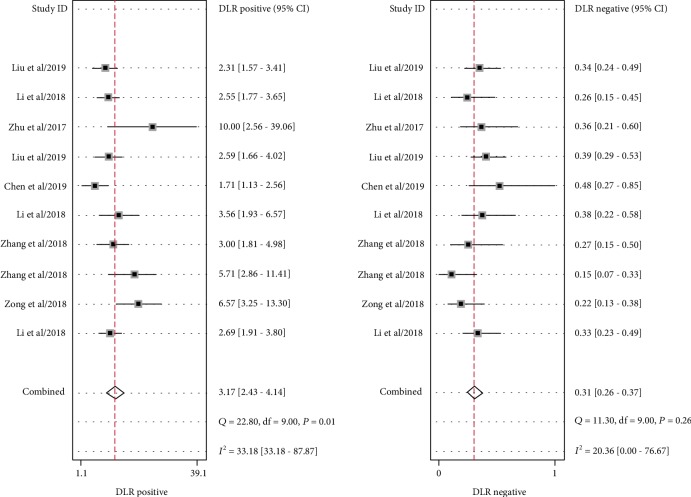
Forest plots of the positive likelihood ratio and the negative likelihood ratio for circRNAs in diagnosing lung cancer.

**Figure 4:**
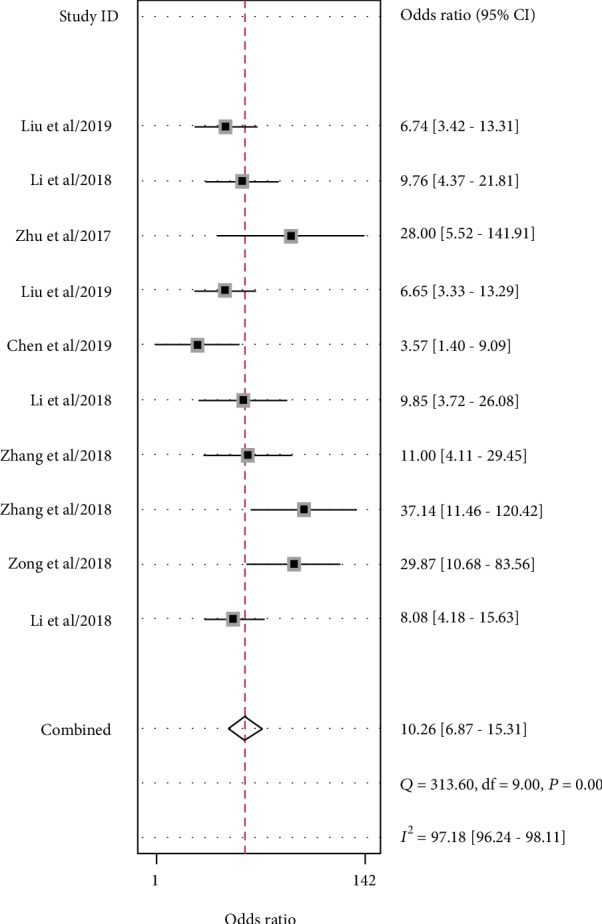
Forest plots of the diagnostic odds ratio for circRNAs in diagnosing lung cancer.

**Figure 5:**
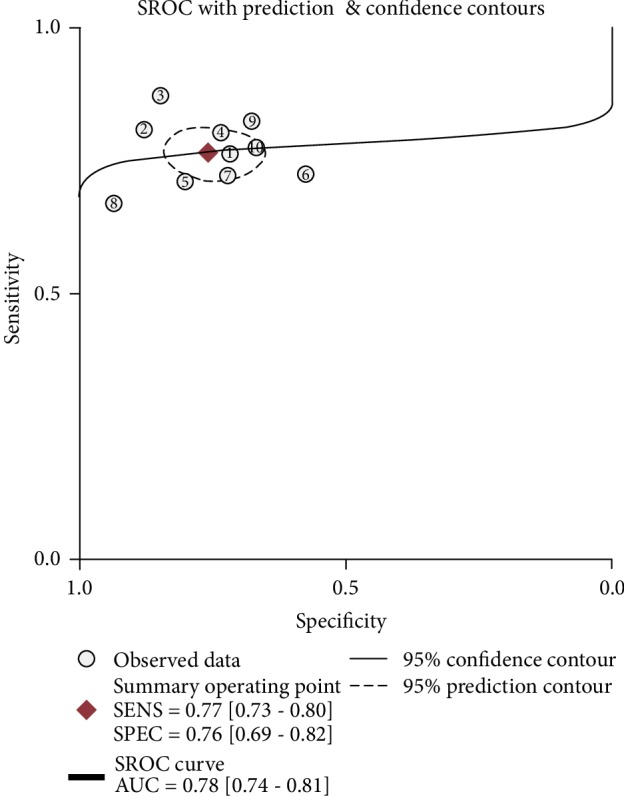
Summary receiver operating characteristic curve for circRNAs in diagnosing lung cancer.

**Figure 6:**
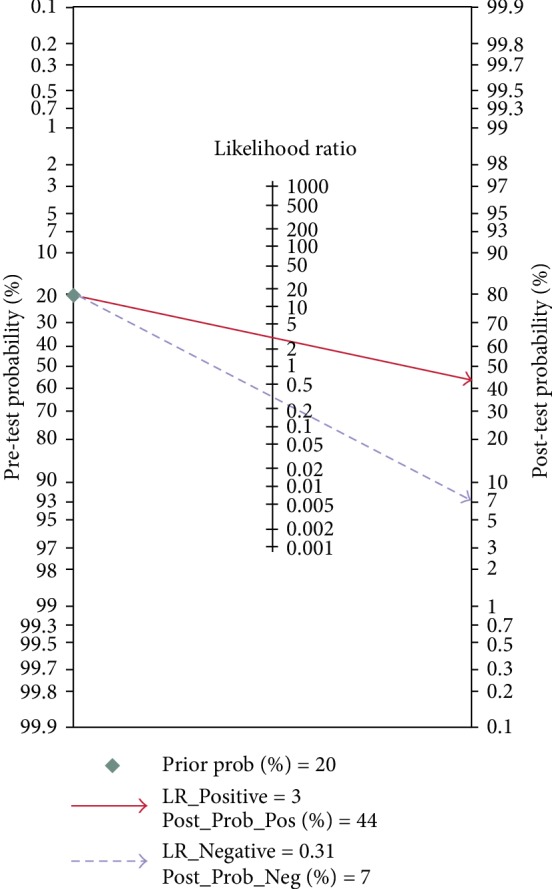
Fagan's nomogram for likelihood ratios.

**Figure 7:**
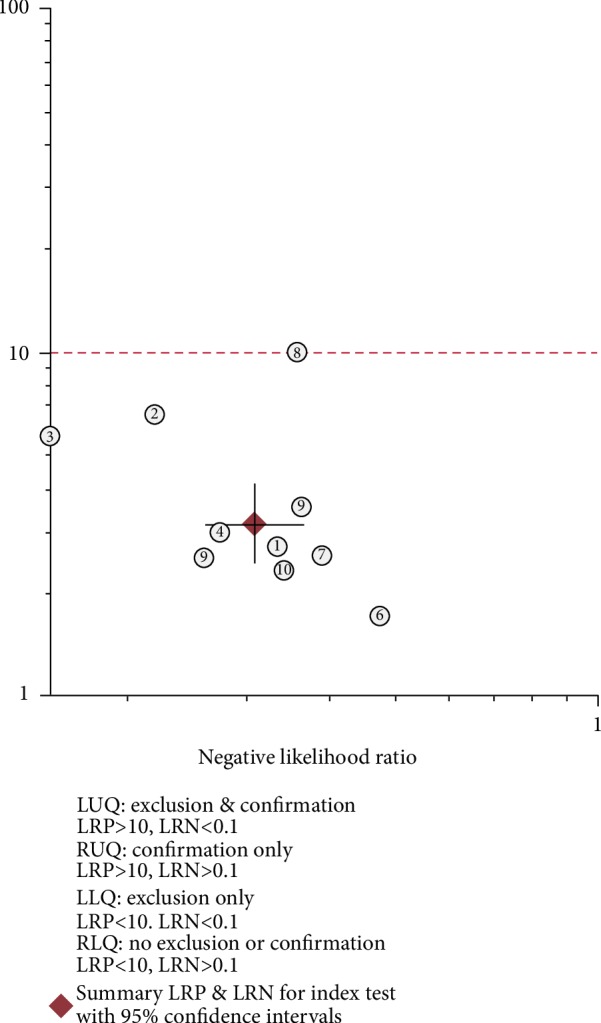
A likelihood ratio scattergram.

Significant heterogeneity was assessed using the random-effects model (*I*^2^ > 50%). For the value of circRNAs in diagnosing lung cancer, the pooled sensitivity, specificity, PLR, NLR, and DOR were 0.77 (95% CI: 0.73–0.80; *I*^2^ = 8.98), 0.76 (95% CI: 0.69–0.82; *I*^2^ = 63.12%), 3.17 (95% CI: 2.43–4.14; *I*^2^ = 33.18%), 0.31 (95% CI: 0.26–0.37; *I*^2^ = 20.36%), and 10.26 (95% CI: 6.87–15.31; *I*^2^ = 97.18%), respectively. AUC was 0.78 (95% CI: 0.74–0.81). Forest plots and SROC are shown in Figures 2–5. Fagan's nomogram is shown in Figure 6. If the pretest probability was 20%, the posttest probability increased to 44%. The pretest likelihood ratio (LR) was 3%, and the posttest LR decreased to 7%. The NLR was 0.31. An LR scattergram was used to evaluate the clinical value of different diagnostic tests and divided into four quadrants (Figure 7). All 10 eligible studies were in the right lower quadrants, suggesting that circRNAs were useful in diagnosing lung cancer.

### 3.4. Regression Analysis

The *I*^2^ value was 96.84%, suggesting significant heterogeneity. The sample type, cancer type, reference gene type, and differential expression of circRNAs were taken as potential causes of heterogeneity, and a metaregression analysis was performed. No significant causes for heterogeneity were found. The results are shown in Table 2.

### 3.5. Subgroup Analysis

A subgroup analysis was performed based on the sample type, sample size, cancer type, reference gene type, and differential expression of circRNAs. Although the sample size did not contribute to heterogeneity, the sensitivity, specificity, and DOR of blood samples were 0.72, 0.78, and 9.32, while the corresponding values of tissue samples were 0.80, 0.75, and 11.67, respectively. These findings suggested that tissue circRNAs were slightly superior to blood circRNAs in diagnosis. The sensitivity, specificity, and DOR of the non-small-cell carcinoma subgroup were 0.78, 0.70, and 9.66, while the corresponding values of the lung adenocarcinoma subgroup were 0.75, 0.80, and 12.54, respectively, suggesting the superiority of circRNAs in lung adenocarcinoma over those in non-small-cell carcinoma. The subgroup analysis of the reference gene was not conducted because only one study considered *β*-actin as the reference gene. The results are shown in Table 3.

### 3.6. Publication Bias

**Figure 8:**
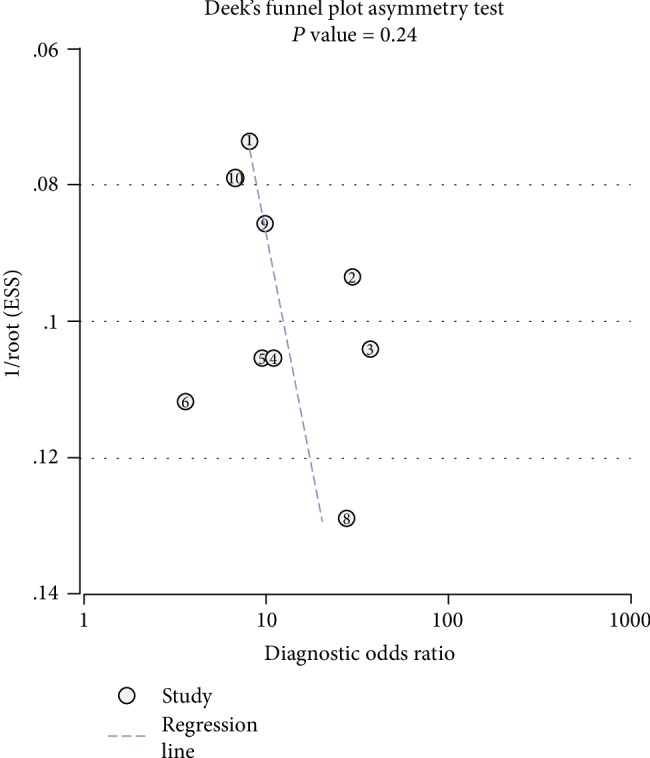
Deeks' funnel plot evaluating the potential publication bias of the included studies.

The publication bias of the included studies was tested using Deeks' funnel plot, and significant differences in the slope rate (*P* < 0.05) suggested the publication bias. The funnel plot was constructed with the Stata 12.0 software (Figure 8), and the results showed no publication bias (*P* = 0.24).

## 4. Discussion

The present meta-analysis enrolled 8 studies from 2017 to 2019 and systemically reviewed 10 circRNAs in diagnosing lung cancer. The results showed that the AUC was 0.78, and the pooled sensitivity, specificity, and DOR were, respectively, 0.77 (95% CI: 0.73–0.80), 0.76 (95% CI: 0.69–0.82), and 10.26 (95% CI: 6.87–15.31). The findings suggested the diagnostic value of circRNAs for lung cancer. The included studies involved only a preliminary analysis of the role of one or two circRNAs in diagnosing lung cancer, with small sample size and sensitivity varying from 0.511 to 0.884 (lower than pooled sensitivity in six studies), specificity from 0.575 to 0.933 (lower than pooled specificity in six studies), and AUC from 0.643 to 0.897 (lower than or equal to AUC in this meta-analysis in five studies). The sensitivity, specificity, and AUC fluctuated largely among these studies, possibly due to the involvement of one or two circRNAs and small sample size.

The Spearman correlation coefficient was calculated to test the threshold effect and was 0.79 (*P* = 0.829), suggesting that the threshold effect was not the cause of heterogeneity. In addition, the regression analysis of sample type, lung cancer type, reference type, and differential expression of circRNAs showed that these factors did not cause heterogeneity. The causes of heterogeneity could not be directly found using the studies included in this analysis. Neither study was a randomized controlled study, possibly leading to heterogeneity. However, this hypothesis should be further verified. Additionally, the present meta-analysis included 8 studies and systematically reviewed the value of 10 different circRNAs in diagnosing lung cancer. The analysis revealed that the expression levels of these circRNAs were different, which might be one of the sources of heterogeneity and also a common problem in this type of analysis.

In conclusion, this systematic review of data extracted from eight studies showed the value of circRNAs in diagnosing lung cancer. These studies were primarily based on the testing of lung cancer tissues. Blood and exocrine secretions were less used, and clinical data were limited. Therefore, the relationship of circRNAs in the blood or exocrine secretions with lung cancer needs further investigation, and the finding might help in the development of molecular markers of diagnosis and prognosis. Further, the recent studies explored the role of a single circRNA in diagnosing and treating cancer using a small sample size; no multicenter and large-sample studies were reported. Hence, the diagnostic accuracy and stability of circRNAs need further elucidation.

## Figures and Tables

**Table 1:** Characteristics of eight studies included in the meta.

Author	Year	circRNAs	Country	Sample type	No. of cases	No. of controls	AUC	Sensitivity	Specificity	Testing method	TNM staging		circRNA expression	References
											I–II	III–IV		
Zhu et al.	2017	hsa_circ_0013958	China	Blood	30	30	0.794	0.667	0.933	qRT-PCR	28	21	Upregulated	[24]
Li et al.	2018	hsa_circ_0079530	China	Tissue	92	92	0.756	0.762	0.721	qRT-PCR	—	—	Upregulated	[15]
Zong et al.	2018	circRNAs_102231	China	Tissue	57	57	0.897	0.812	0.887	qRT-PCR	30	27	Upregulated	[16]
Zhang et al.	2018	hsa_circ_0014130	China	Tissue	46	46	0.878	0.87	0.848	qRT-PCR	36	10	Upregulated	[17]
Zhang et al.	2018	circ_FOXO3	China	Tissue	45	45	0.782	0.8	0.733	qRT-PCR	—	—	Downregulated	[18]
Li et al.	2018	circ_PVT1	China	Blood	45	45	0.794	0.711	0.8	qRT-PCR	31	37	Upregulated	[19]
			China	Tissue	68	68	0.803	0.825	0.675	qRT-PCR	31	37	Upregulated	
Chen et al.	2019	circRNAs_100146	China	Tissue	40	40	0.643	0.725	0.575	qRT-PCR	—	—	Upregulated	[20]
Liu et al.	2019	hsa_circ_0005962	China	Blood	153	54	0.73	0.719	0.7222	qRT-PCR	96	55	Upregulated	[21]
		hsa_circ_0086414	China	Blood	153	54	0.78	0.7712	0.6667	qRT-PCR	96	55	Downregulated	

Note: data not extracted; AUC: area under the receiver operating characteristic curve.

**Table 2:** Relative diagnostic odds ratio of covariants in the metaregression analysis.

	RDOR	95% CI	*P* value
Sample type	2.07	(0.62–6.95)	0.1710
Cancer type	1.38	(0.41–4.67)	0.5064
Reference	0.27	(0.04–1.63)	0.1125
Expression of circRNAs	0.81	(0.21–3.18)	0.6918

**Table 3:** Summary results of the subgroup analysis for circRNAs in diagnosing lung cancer.

	Number of studies	Sensitivity (95% CI)	Specificity (95% CI)	PLR (95% CI)	NLR (95% CI)	DOR (95% CI)	AUC
Sample size							
Blood	4	0.72 (0.66–0.78)	0.78 (0.66–0.87)	3.30 (2.12–5.15)	0.35 (0.29–0.43)	9.32 (5.35–16.23)	0.78
Tissue	6	0.80 (0.75–0.84)	0.75 (0.66–0.82)	3.16 (2.22–4.49)	0.27 (0.20–0.36)	11.67 (6.36–21.39)	0.84
Lung cancer							
Non-small-cell lung cancer	6	0.78 (0.73–0.83)	0.70 (0.66–0.79)	2.87 (2.22–3.72)	0.30 (0.23–0.38)	9.66 (6.00–15.55)	0.82
Lung adenocarcinoma	4	0.75 (0.69–0.79)	0.81 (0.68–0.90)	3.93 (2.20–7.03)	0.31 (0.25–0.40)	12.54 (5.96–26.39)	0.76
Reference gene							
GAPDH	9	0.76 (0.72–0.79)	0.75 (0.68–0.81)	3.00 (2.31–3.90)	0.33 (0.28–0.38)	9.41 (6.66–13.31)	0.77
circRNA expression upregulation	8	0.76 (0.72–0.80)	0.77 (0.69–0.84)	3.38 (2.39–4.77)	0.31 (0.25–0.38)	10.96 (6.57–18.29)	0.79

Note: PLR: positive likelihood ratio; NLR: negative likelihood ratio; AUC: area under the receiver operating characteristic curve.
